# A meta-analysis of the efficacy of balloon dilation intervention for patients with cricopharyngeal achalasia after stroke

**DOI:** 10.3389/fneur.2026.1797311

**Published:** 2026-09-09

**Authors:** Lingyun Wu, Weifeng Jiang

**Affiliations:** 1 School of Medical Sciences, Quzhou College of Technology, Quzhou, China; 2 Department of Geriatric Medicine, Quzhou People’s Hospital; The Quzhou Affiliated Hospital, Wenzhou Medical University, Quzhou, China

**Keywords:** balloon dilatation, cricopharyngeal achalasia, meta-analysis, stroke, swallowing disorders

## Abstract

**Background:**

The present study aimed to evaluate the efficacy and safety of balloon dilatation for the treatment of swallowing disorders after stroke.

**Methods:**

PubMed, Embase, the Cochrane Library, Web of Science, China National Knowledge Infrastructure (CNKI), VIP, and the Chinese Wanfang database were searched from their inception to 30 October 2024. Two authors independently screened all studies, extracted data, and evaluated the risk of bias in the included studies. Review Manager 5.4 and Stata software (version 16) were used to assess study quality.

**Results:**

A total of 25 studies involving 1,134 patients were included in the present analysis. The results suggest that balloon dilatation significantly improves the effective rate (OR = 6.11; 95% CI: 4.16, 8.98), videofluoroscopic swallowing study (VFSS) score (MD = 2.23, 95% CI: 1.98, 2.48), and Functional Oral Intake Scale (FOIS) score (MD = 1.93, 95% CI: 1.40, 2.47); it also shortens swallowing passage time (MD = −0.07; 95% CI: −0.08, −0.06), improves the Kubota Drinking Water Test (KDWT) score (MD = −1.01, 95% CI: −1.55, −0.47), and reduces the incidence of aspiration pneumonia (OR = 0.46; 95% CI: 0.21, 0.98).

**Conclusion:**

Compared to routine rehabilitation training, balloon dilation is a safer and more effective method for improving swallowing function.

## Introduction

Cricopharyngeal achalasia (CPA), a prevalent complication in stroke patients, is characterized by delayed relaxation or increased resting tone of the cricopharyngeus muscle during deglutition (1–3). It leads to upper esophageal sphincter dysfunction manifested as absent, incomplete, or discoordinated opening, and its worldwide prevalence reaches 65% (2–4). CPA can lead to serious complications, such as dehydration, aspiration pneumonia, and malnutrition, all of which can increase the risk of poor prognosis and mortality rates (3, 5, 6). Effective intervention plays a pivotal role in the clinical treatment of CPA. Therapeutic approaches for CPA have gradually evolved following the recognition of the condition. Initially, management depended on surgical myotomy and bougie dilatation, however, over time, conservative rehabilitation and botulinum toxin injections have emerged as minimally invasive alternatives (7–13). Balloon dilatation has undergone notable technical advancements: Rigid bougies have been superseded by inflatable devices, and pneumatic balloons have been optimized into adjustable water-filled catheters (14–16). Fluoroscopy-guided transnasal balloon dilatation is now widely used in post-stroke CPA treatment. As an innovative strategy for swallowing rehabilitation, balloon dilatation demonstrates advantages including safety, efficacy, few adverse events, and cost-effectiveness (15–17). Although many studies have demonstrated that balloon dilation can effectively ameliorate post-stroke swallowing disorders while mitigating adverse reactions such as mucosal edema, injury, bleeding, and pain (15, 18, 19), the results have been inconsistent. Some scholars argued that balloon expansion training may not effectively stimulate the brain to elicit the swallowing nerve reflex, thereby failing to achieve the anticipated therapeutic outcome (20–22). Thus, this study aimed to evaluate the efficacy and safety of balloon dilation in patients with post-stroke dysphagia to generate more rigorous evidence to support its clinical application. Compared with previous meta-analyses, we retrieved updated evidence from both Chinese and English databases, synthesizing multiple swallowing function indicators alongside the safety endpoint of aspiration pneumonia incidence. This comprehensive safety assessment enables our study to yield balanced evidence covering both therapeutic efficacy and clinical safety.

## Methods

### Literature search

We followed the Preferred Reporting Items for Systematic Reviews and Meta-Analyses (PRISMA) guidelines as a quality control procedure for the selection of articles. To comprehensively obtain relevant literature, a systematic strategy was utilized by two authors to independently search for relevant studies published in China National Knowledge Infrastructure (CNKI), VIP, Wanfang Database, PubMed, Embase, the Cochrane Library, and Web of Science up to 30 October 2024, using the keywords (“stroke” OR “poplexy” OR “cerebrovascular stroke” OR “cerebral stroke”) AND (“deglutition disorders” OR “dysphagia” OR “swallowing disorder”) AND (“catheterization” OR “dilatation” OR “balloon dilatation”). No restriction was imposed on date, language, or publication status. We checked the reference lists of the identified studies and review articles for additional potentially eligible records. The search strategies are provided in Supplementary Table 1.

### Inclusion and exclusion criteria

The studies that were eligible for inclusion met the following criteria: (1) they were randomized controlled trials (RCTs); (2) patients were confirmed to have had a stroke by CT or MRI; (3) patients were diagnosed with CPA through a clinical swallowing evaluation, which was then further confirmed by a videofluoroscopic swallowing study (VFSS); (4) patients received either balloon dilatation or routine rehabilitation training; and (5) at least one of the following outcome measures was reported: effective rate of treatment, swallowing passage time, VFSS score, Functional Oral Intake Scale (FOIS) score, Kubota Drinking Water Test (KDWT) score, and incidence of aspiration pneumonia. The exclusion criteria for the article were as follows: (1) articles with insufficient data, (2) low-quality articles, and (3) duplicate articles.

### Data extraction and quality assessment

Two independent researchers extracted data from the included studies, which were cross-checked and adjudicated by a third researcher in case of any disputes. The collected data consisted of first author, publication year, country or region, sample size, number of cases, treatment interventions, intervention time, and outcome measures.

### Statistical analysis

Review Manager (version 5.4) (RevMan, The Cochrane Collaboration, Oxford, United Kingdom) and Stata 16 (StataCorp, College Station, TX, United States) were used to analyze the data in this meta-analysis. When the included studies were homogeneous (*p* ≥ 0.1, *I*^2^ ≤ 50%), a fixed-effects model was used for the analysis. Otherwise, a random-effects model was used. A sensitivity analysis was also conducted to assess heterogeneity. Publication bias was assessed qualitatively using funnel plots and quantitatively through Begg’s test and Egger’s test. All *p*-values were two-sided, with a *p*-value of <0.05 indicating statistical significance.

## Results

### Search results and study characteristics

**Figure 1:**
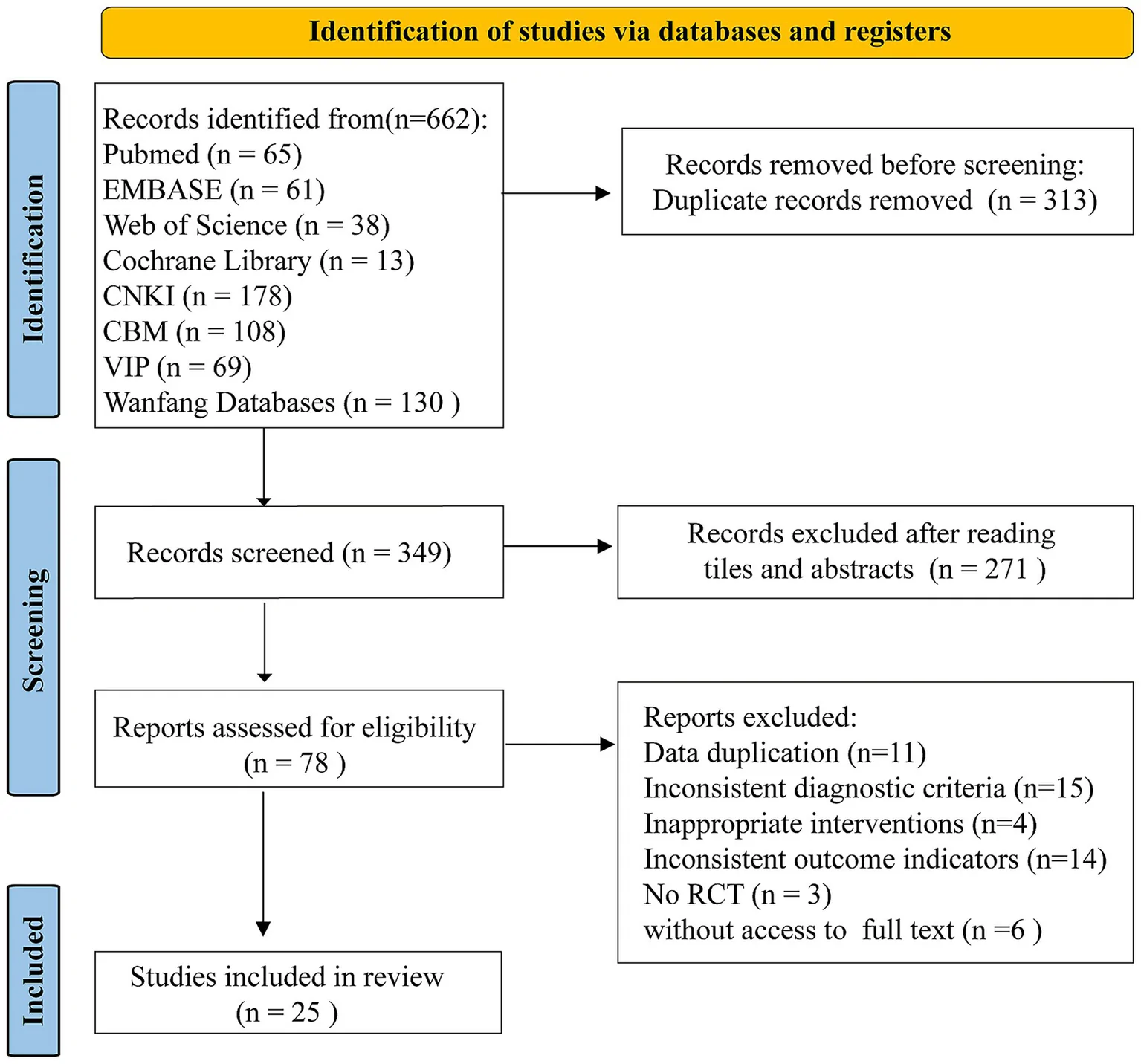
Flow diagram of search strategy and study selection.

As shown in Figure 1, 662 studies were identified with a computerized search, 313 were duplicate articles, and 271 were excluded after reviewing the title and abstract. Then, 78 potentially relevant articles were selected for full-text review, and 6 articles did not meet our inclusion criteria. Finally, 25 studies involving 1,134 patients that met our inclusion criteria were eligible to be included in the qualitative synthesis. A comprehensive explanation of all the included studies is presented in Table 1. In these studies, all studies were carried out in China, involving 1,134 subjects, 570 in the experimental group and 564 in the control group, and the reported time of intervention varied from 2 to 6 weeks. Similarly, we tabulated the number of studies and patient samples included for different interventions.

**Table 1:** Characteristics of included studies.

Author	Year	Country	Sample Size		Age		Sex (M/F)		Symptom Duration		Intervention period	Clinical Efficacy
			EG	CG	EG	CG	EG	CG	EG	CG		
Chen MM (33)	2020	China	48	48	64.45 ± 5.46	63.89 ± 5.07	29/19	30/18	NA	NA	4 w	ER, AP
Ding HY (34)	2021	China	31	31	55.36 ± 4.49	54.23 ± 4.31	18/13	16/15	2.54 ± 0.68 w	2.62 ± 0.57 w	4w	ER, VFSS, FIOS, KDWT, AP
Dou ZL (35)	2013	China	15	15	59.09 ± 12.97	58.36 ± 11.99	12/3	11/4	5.45 ± 4.48 m	5.27 ± 3.52 m	3w	FIOS
Feng JJ (36)	2017	China	19	19	49.6 ± 7.9	51.9 ± 8.1	12/7	14/5	14.1 ± 2.0d	15.0 ± 2.2d	5w	VFSS, KDWT
Gao XC (37)	2018	China	21	20	57.6 ± 7.3	59.8 ± 6.7	15/6	12/8	2.6 ± 1.7 m	2.4 ± 1.9 m	4w	SPT, VFSS, FIOS
He WN (38)	2017	China	31	30	59.00 ± 8.72	58.50 ± 8.85	13/18	16/14	131.40 ± 29.80d	131.55 ± 28.34d	30d	ER, VFSS, KDWT
He YG (39)	2016	China	16	16	65.36 ± 8.67	66.75 ± 7.13	11/5	12/4	2.61 ± 2.74 m	2.32 ± 2.88 m	2w	ER, SPT, VFSS
Huang SC (40)	2016	China	19	19	61 ± 11	63 ± 15	12/7	10/9	4.2 ± 2.7 m	3.8 ± 2.4 m	4w	ER, VFSS
Huang XH (23)	2017	China	20	20	60.5 ± 3.4	60.8 ± 3.1	12/8	13/7	35.6 ± 10.9d	35.2 ± 10.7d	3w	ER, VFSS, FIOS, SPT
Lan Y (18)	2013	China	15	15	62(47–66)	60(50–69)	12/3	11/4	4(3-8)m	4(3-7)m	3w	ER, FIOS
Lan Y (41)	2009	China	15	15	51.0 ± 13.6	50.0 ± 10.7	10/5	9/6	3.2 ± 2.1 m	3.7 ± 1.9 m	4w	ER, SPT
Lin M (42)	2018	China	25	25	52.6 ± 5.5	51.4 ± 4.9	14/11	15/10	NA	NA	4w	ER, VFS, FIOS
Liu LR (43)	2016	China	10	10	60(52–65)	59(50–64)	6/4	5/5	NA	NA	10d	ER, VFSS
Shao WB (44)	2017	China	32	32	61.09 ± 6.82	62.78 ± 7.08	21/11	22/10	NA	NA	4w	ER
Tang GH (45)	2017	China	44	44	51.1 ± 10.5	51. 3 ± 10. 4	28/16	29/15	NA	NA	6w	ER, VFSS, SPT
Wang GM (46)	2016	China	22	22	NA	NA	NA	NA	NA	NA	7d	ER, FIOS
Wang J (47)	2014	China	16	16	63.15 ± 6.32	65.9 ± 57.06	11/5	13/3	19.67 ± 7.68d	20.98 ± 6.51d	3w	ER
Yu Y (48)	2013	China	18	14	NA	NA	NA	NA	NA	NA	15d	ER, SPT
Zhang BX (49)	2018	China	16	16	63.56 ± 11.09	65.06 ± 8.03	10/6	14/2	2.72 ± 2.88 m	3.01 ± 2.44 m	6w	ER, VFSS
Zhang FW (50)	2017	China	15	15	53.13 ± 6.44	54.62 ± 5.98	8/7	9/6	5.56 ± 1.32 m	5.75 ± 1.66 m	4w	FIOS
Zhang F (51)	2021	China	15	15	65.47 ± 5.33	65.23 ± 5.12	10/5	9/6	2.26 ± 0.15 m	2.11 ± 0.12 m	NA	ER
Zhang WW (52)	2020	China	19	19	65.89 ± 12.13	68.34 ± 7.56	12/7	11/8	3.02 ± 1.68 m	2.86 ± 1.45 m	2w	KDWT
Zhou HY (53)	2019	China	30	30	58. 65 ± 5. 33	59.02 ± 5.29	18/12	20/10	NA	NA	4w	ER, VFSS
Zhou WJ (54)	2021	China	33	33	61.79 ± 12.85	66.30 ± 10.26	17/16	14/19	6.01 ± 3.57 m	6.06 ± 3.52 m	3w	SPT, FIOS
Zhu HX (55)	2018	China	25	25	58. 65 ± 5. 33	58.96 ± 11.62	15/10	14/11	45.0 ± 23.8d	40.0 ± 25.1d	4w	ER, VFSS, FIOS, SPT, AP

EG, experimental group; CG, control group; M, male; F, female; NA, not applicable; m, months; w, weeks; D, days; ER, effective rate; SPT, swallowing passage time; KDWT, Kubota drinking water test; VFSS, video fluoroscopy swallowing study; FOIS, Functional Oral Intake Scale; AP, aspiration pneumonia. The control group underwent conventional rehabilitation training, including routine care and swallowing training. The experimental group received balloon dilation in addition to the above regimen.

### Risk assessment and methodological quality evaluation

**Figure 2:**
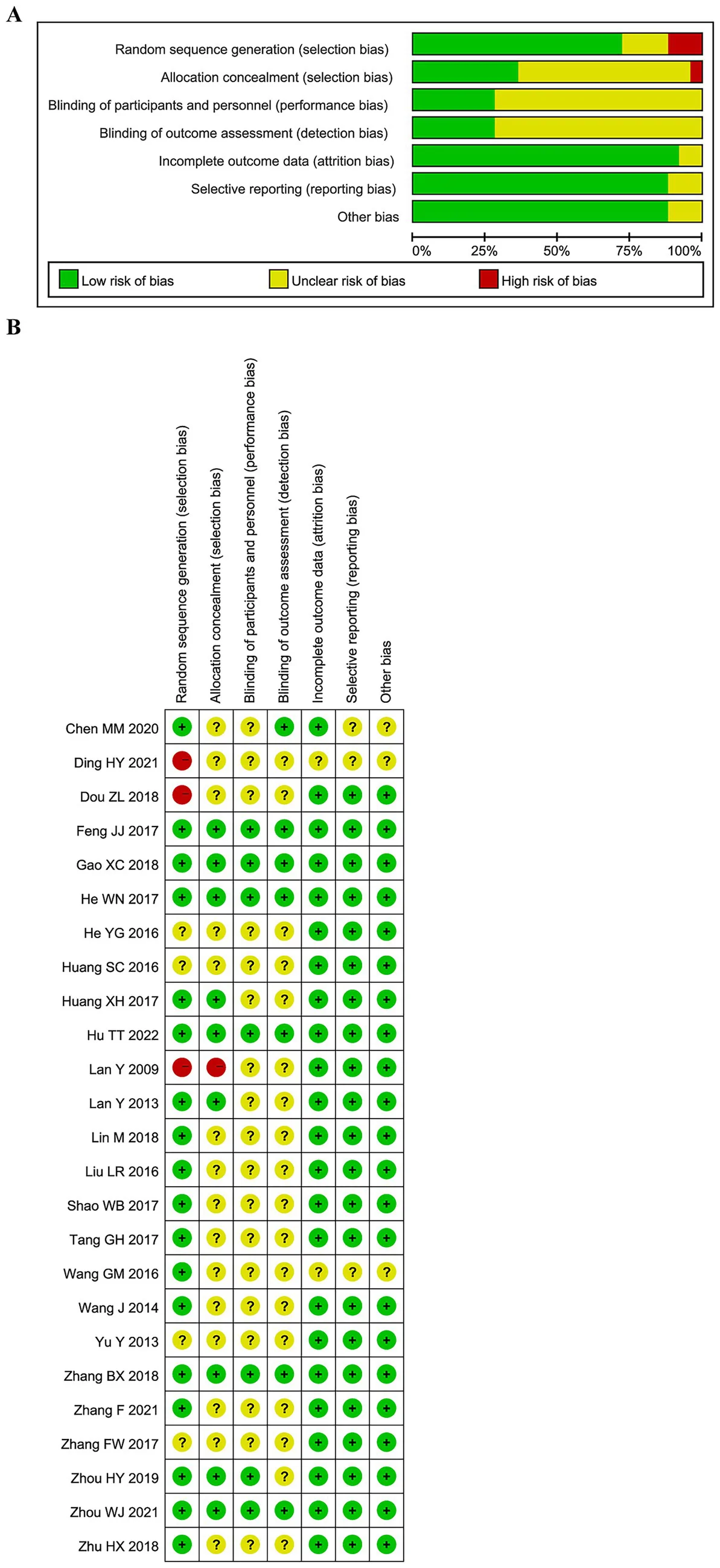
Quality assessment for each eligible study was summarized in **(A)** as percentages across all included studies in the risk of bias chart or **(B)** in risk of bias summaries. red (−): High risk of bias, yellow (?): Bias risk unclear, green (+): Low bias risk.

Two researchers independently assessed the risk of bias and methodological quality of all included articles. Regarding the risk of bias tool, among the 25 included studies, 18 described their randomization methods, using a random number table to assign groups, and were assessed as having a low risk of bias. Four studies did not describe the concrete randomization method, thus resulting in an unclear risk of bias. However, the remaining articles did not mention the selection methods, thus resulting in a high risk of bias. Due to the nature of the interventions, it is difficult for researchers to implement blinding. Only 7 studies managed to blind participants and researchers in the intervention process, and 23 studies mentioned blinding for outcome measurers. For other biases, all articles presented the statistical homogeneity of the baseline data and were evaluated as having a “low risk” of bias. The risk assessment details for the included articles are shown in Figure 2.

### Results of the effective rate of treatment

**Figure 3:**
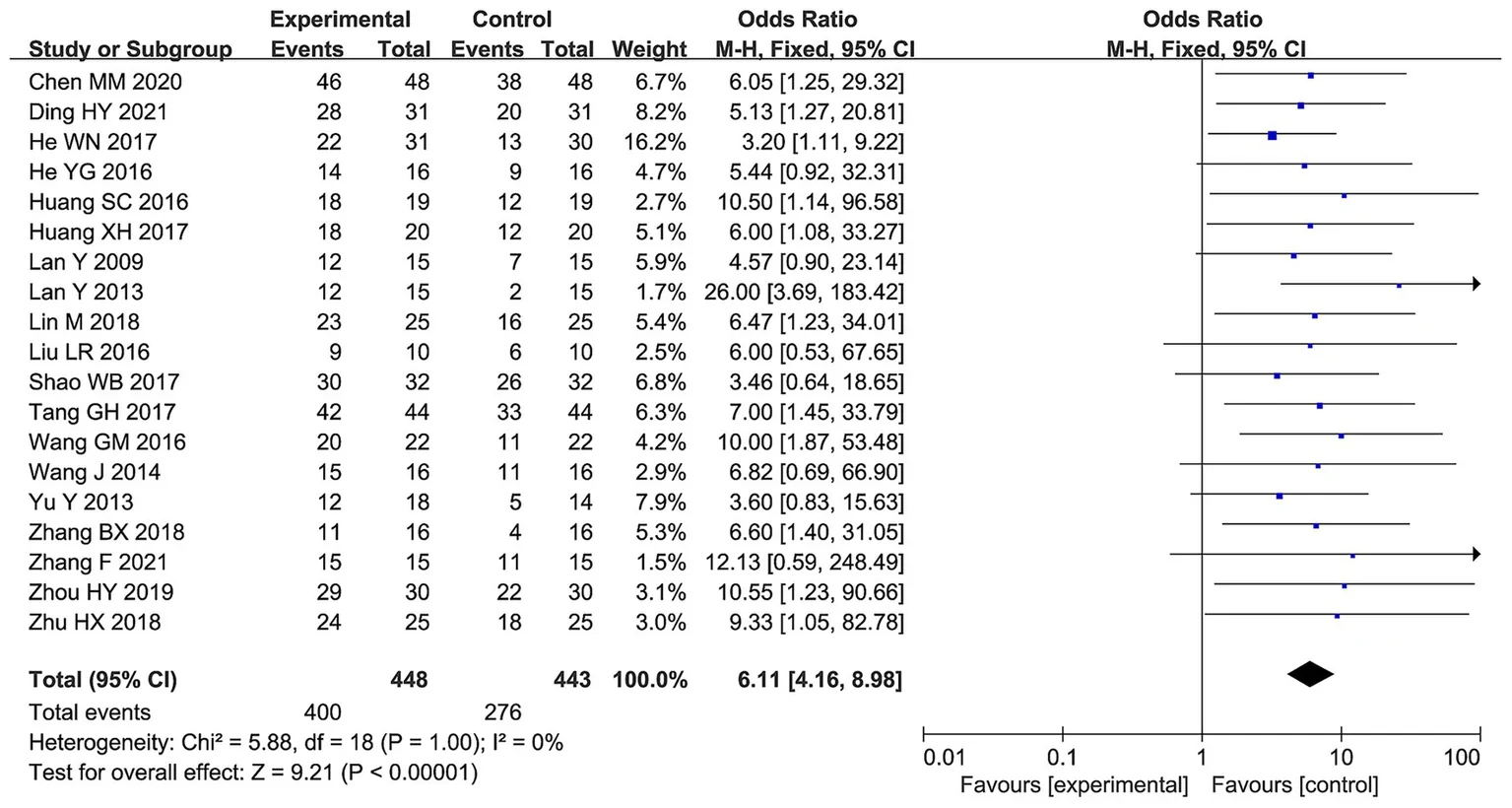
Forest plot for the effective rate.

Among the 25 included articles, 19 reported the treatment effectiveness rate, and there was no heterogeneity among the studies (*p* = 1.00, *I*^2^ = 0%). Using the fixed-effect model, the analysis results showed that the effective rate of treatment of swallowing disorders (OR = 6.11; 95% CI: 4.16, 8.98) in the experimental group was better than that in the control group; the difference between groups was statistically significant (*p* < 0.05, Figure 3).

### Results of the swallowing passage time changes

**Figure 4:**
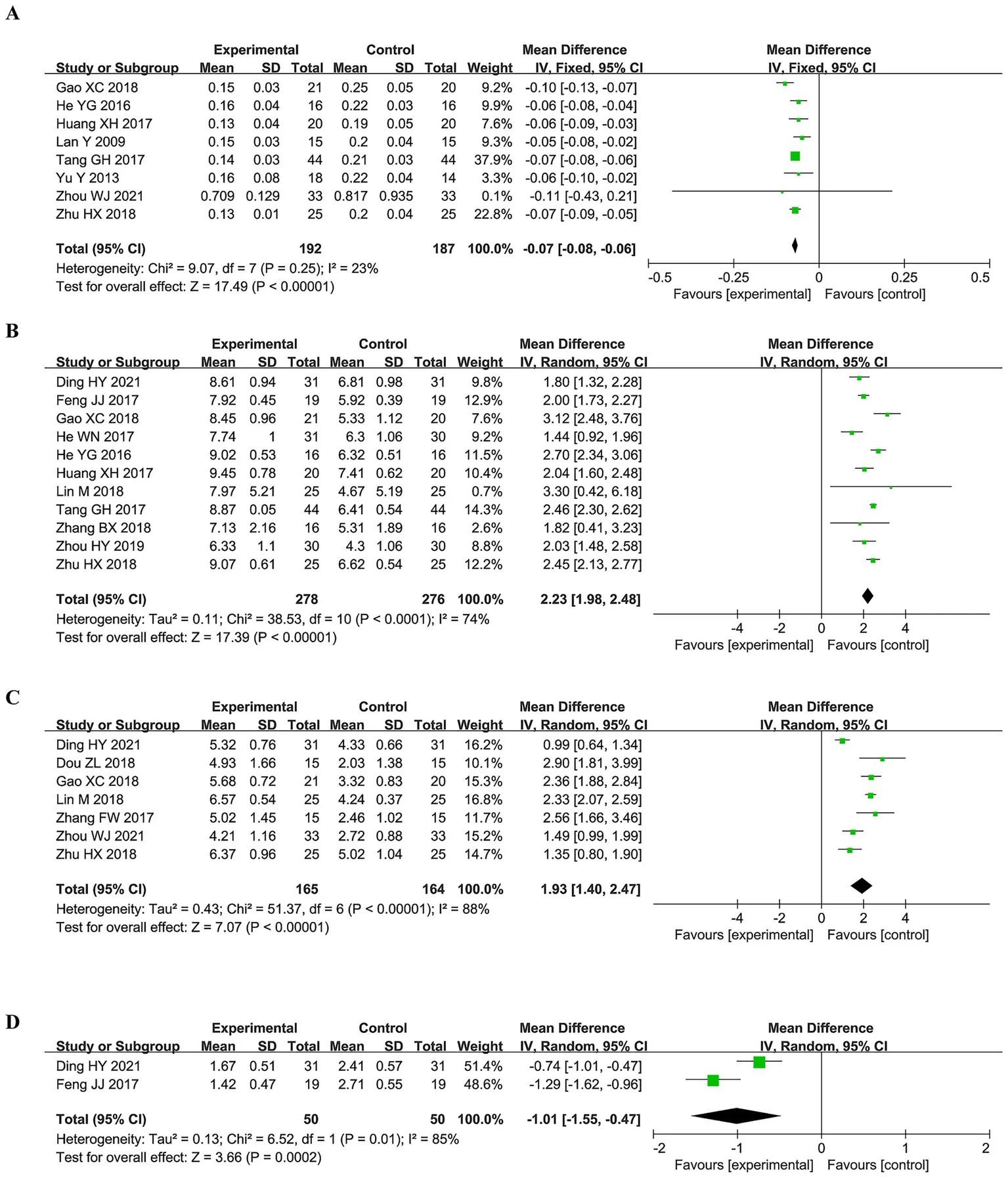
Forest plot for the swallowing function: **(A)** Swallowing passage time, **(B)** VFSS, **(C)** FIOS, and **(D)** KDWT.

Among the 25 included articles, 8 provided complete swallowing passage time data before and after treatment. Our results showed that the swallowing passage time in the experimental group was significantly lower than that in the control group (MD = −0.07; 95% CI: −0.08, −0.06), as shown in Figure 4A.

### Results of the VFSS score changes

Eleven studies reported the VFSS score. The VFSS score in the experimental group was significantly higher than that in the control group (MD = 2.23, 95% CI: 1.98, 2.48, Figure 4B).

### Results of the FOIS score changes

Eight studies were initially included in this analysis; however, the results of Huang et al. were excluded, as they were inconsistent with the description of their findings (23). Finally, seven studies were included, revealing significant heterogeneity (*p* < 0.0001, *I*^2^ = 88%). Consequently, a random-effects model was used to account for the heterogeneity. The results demonstrated that the FOIS score in the experimental group was higher than in the control group (MD = 1.93, 95% CI: 1.40, 2.47; Figure 4C).

### Results of the KDWT score changes

Two studies directly reported the KDWT score. A random-effects model was applied because of strong heterogeneity among the two studies (*I*^2^ = 85%, *p* = 0.01). As shown in Figure 4D, the KDWT score of the observation group is lower than that of the control group (MD = −1.01, 95% CI: −1.55, −0.47).

### The incidence of aspiration pneumonia

**Figure 5:**
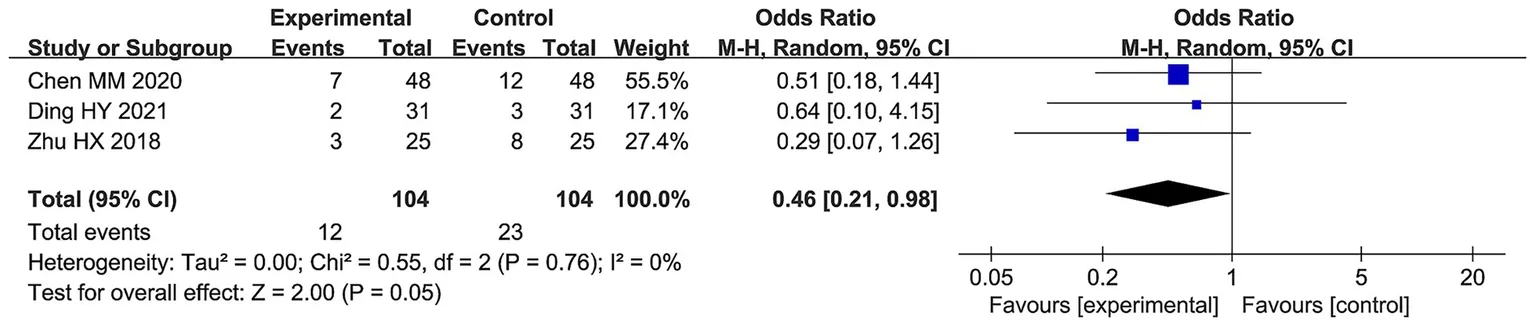
Forest plot for aspiration pneumonia.

The most common complication reported in the trials was aspiration pneumonia. Three studies reported, and pooled results are shown in Figure 5. The incidence of aspiration pneumonia in the experimental group was lower than that in the control group (*I*^2^ = 0%; OR, 0.46; 95% CI: 0.21, 0.98).

### Sensitivity analysis

For significant heterogeneous outcomes (VFSS, FIOS, KDWT score), as indicated by *I*^2^ > 50% and *p* < 0.10 in the heterogeneity test, sensitivity analysis was performed. The effect sizes of the random-effects and fixed-effects models were identical (Table 2).

**Table 2:** Sensitivity analysis results.

Outcome measure	Model	MD	95% confidence interval		*I*^2^ (%)	*p*
			Lower limit	Upper limit		
VFSS	Random	2.23	1.98	2.48	74	<0.0001
	Fixed	2.31	2.20	2.41	74	<0.0001
FIOS	Random	1.93	1.40	2.47	88	<0.0001
	Fixed	1.89	1.72	2.05	88	<0.0001
KDWT	Random	−1.01	−1.55	−0.47	85	0.01
	Fixed	−0.96	−1.17	−0.76	85	0.01

VFSS, video fluoroscopy swallowing study; FOIS, Functional Oral Intake Scale; KDWT, Kubota drinking water test.

### Publication bias analysis

**Figure 6:**
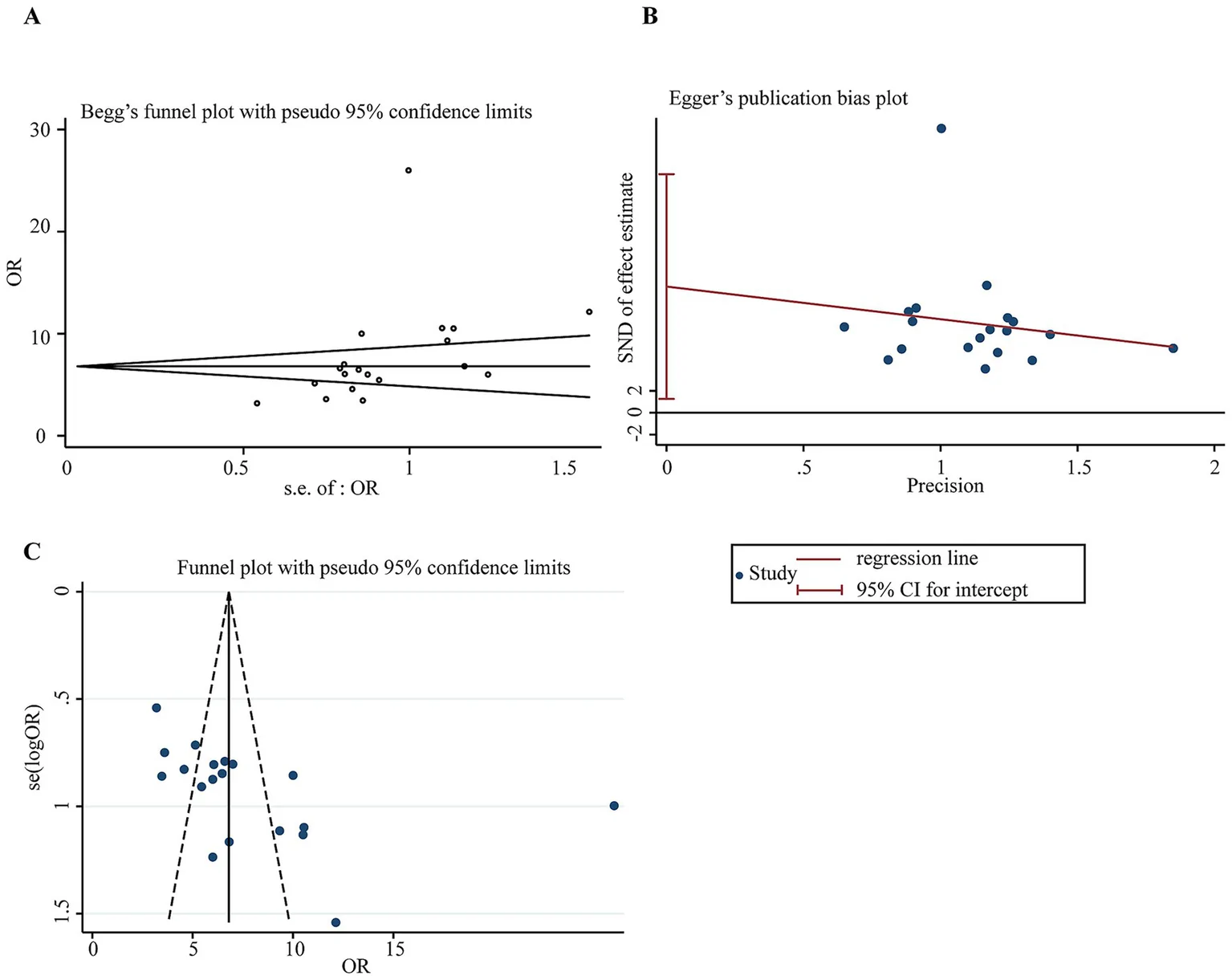
Publication bias analysis of the effect rate: **(A)** Begg’s test, **(B)** Egger’s test, and **(C)** Funnel plot.

As shown in Figure 6, Egger’s (*t* = 2.37, *p* = 0.03) and Begg’s (*p* = 0.017, Z = 2.38) tests showed significant evidence of publication bias for the effective rate. Consistently, the funnel plot also showed asymmetry.

## Discussion

Cricopharyngeal achalasia (CPA), a key contributor to post-stroke dysphagia, is a neuromuscular disorder of the upper esophageal sphincter (UES) marked by incomplete or delayed relaxation of the cricopharyngeus muscle during swallowing (1, 6). Current clinical strategies for CPA include rehabilitation training, neuromuscular electrical stimulation, balloon dilation, cricopharyngeal myotomy, and combined therapeutic regimens (8, 9, 11, 12). These interventions can improve swallowing function. While universal first-line therapies for CPA remain undefined, balloon dilatation has been validated for efficacy, feasibility, and patient acceptability. The central findings of our meta-analysis indicated that balloon dilatation for patients with post-stroke dysphagia improve swallowing function more than conventional rehabilitation training, which includes swallowing and feeding training, breathing training, and related exercises. Balloon dilatation can rapidly relieve functional obstruction caused by CPA and achieve immediate improvements in UES opening and swallowing performance (4). However, some clinical observations indicate that the short-term benefits may not persist sustainably. For some patients, swallowing dysfunction recurs gradually within several months after treatment, thereby requiring periodic maintenance dilatation to sustain therapeutic effects (24, 25). This limitation highlights the vulnerability of balloon dilatation as monotherapy. Encouragingly, accumulating evidence suggests that combined interventions integrating balloon dilatation with rehabilitation training, neuromuscular electrical stimulation, or botulinum toxin type A injection may help prolong the duration of efficacy and lower recurrence risks (11, 12). Future research should focus on exploring individualized combination protocols to achieve persistent recovery of swallowing function among post-stroke CPA populations.

Balloon dilatation is an endoscopic pneumatic dilation treatment for rupture of the muscle fibers of the lower esophageal sphincter, in which dilatation of an inflated balloon is performed to reduce the tension caused by the rupture (26, 27). Clinical efficacy and swallowing function can be evaluated either quantitatively by measuring swallow passage time and FOIS, or instrumentally via VFSS. The results of the present study showed that the observation group had more advantages in clinical effectiveness rate than the control group. The VFSS and FOIS scores in the observation group with conventional swallowing rehabilitation combined with balloon dilatation were better than those of the control group with conventional swallowing rehabilitation alone. Beyond the conventional swallowing function indicators used in previous meta-analyses, we also included KDWT outcomes. The KDWT score is lower than that of the control group, suggesting that balloon dilatation in CPA patients has a clear therapeutic effect on swallowing disorders caused by cricopharyngeal muscle atonia after stroke compared with conventional swallowing rehabilitation. Since balloon dilatation is not a mechanical pull on the cricopharyngeal muscle, it can strengthen the cricopharyngeal muscles and restore central nerve conduction to the cricopharyngeal muscles by enlarging the entrance to the esophagus, and ultimately facilitate swallowing function and shorten the recovery time (16, 28, 29). Existing evidence suggests that the efficacy of balloon dilatation is affected by the balloon catheter itself and its operation, such as material and structure (19). Given the limited number of included trials, this meta-analysis did not distinguish differences in balloon catheter type, dilatation routes, timing of intervention, and other possible influences.

Pneumonia an early complication of stroke and occurs in approximately 10% of hospitalized patients in the first days of the illness (30, 31). Especially in CPA patients, the incidence of pneumonia may be as high as 40% (31). Only three studies that reported the occurrence of aspiration pneumonia were included in this study, and the results showed that balloon dilation can significantly reduce the incidence of aspiration pneumonia in patients with dysphagia after stroke. Our results indicated that balloon dilation is helpful for relieving the symptoms of stroke patients complicated by dysphagia caused by cricopharyngeal achalasia, with relatively high safety. However, the result was inconsistent with the results of Zhuang et al. (32). This may be due to the small sample size. Therefore, more high-quality, large-sample-size RCTs are needed in the future to validate the incidence of aspiration pneumonia in patients with CPA.

Although almost all included studies described comparability at baseline, demographic and methodological characteristics such as diagnostic criteria for stroke dysphagia, inclusion and exclusion criteria, interventions, and outcome metrics were not consistent, all of which may be a source of heterogeneity. Although our results were found to be relatively robust through sensitivity analysis, we need to further explore the effects of the above confounding factors on the efficacy of the intervention in larger-sample RCTs with a greater degree of rigor.

The present study has several limitations. All included studies were performed in China. In addition, most researchers reported only the randomization process, not blinding, which may bias the results. Balloon dilatation is a safe intervention when used correctly, but therapists should be aware of its risks in certain areas. However, the numbers are too small to identify important differences in some of the most common complications, such as aspiration pneumonia, dehydration, and malnutrition. The frequency of interventions in the included studies was inconsistent, and the selection of balloon catheters varied, which may have led to a reduction in the efficacy of the test. Moreover, the long-term efficacy could not be examined due to the lack of follow-up. Additionally, this review was not prospectively registered, which may have reduced the transparency of the predefined methodology and any changes made during the review process.

## Conclusion

The current meta-analysis concludes that compared with routine rehabilitation training, balloon dilation is more effective in stroke patients who are undergoing cricopharyngeal achalasia, improving swallowing function and reducing the incidence of aspiration pneumonia, which is worthy of clinical promotion and application. Future randomized clinical trials with larger sample sizes and long-term follow-up are needed to confirm these results.

## Data Availability

The original contributions presented in the study are included in the article/Supplementary material, further inquiries can be directed to the corresponding author.
