# Supplementary material for: A meta-analysis of the efficacy of balloon dilation intervention for patients with cricopharyngeal achalasia after stroke
Source: Front Neurol. 2026 Sep 9;17:1797311. doi: 10.3389/fneur.2026.1797311 (PMC13597724; doi:10.3389/fneur.2026.1797311)
Supplement: Supplementary file 1 [file Table_1.DOCX]

**Supplementary Table1.** Search strategies

PubMed

#1:"Stroke"[Mesh] OR "poplexy"[Title/Abstract] OR "cerebrovascular stroke"[Title/Abstract] OR "cerebral stroke"[Title/Abstract]

#2:"Deglutition Disorders"[Mesh] OR "dysphagia"[Title/Abstract] OR "swallowing disorder"[Title/Abstract]

#3:"Catheterization"[Mesh] OR "dilatation"[Title/Abstract] OR "balloon dilatation"[Title/Abstract]

#4: #1 AND #2 AND #3
